# Predictors of Mental Health Status among Older United States Adults with Pain

**DOI:** 10.3390/bs11020023

**Published:** 2021-02-05

**Authors:** David R. Axon, Jonathan Chien

**Affiliations:** College of Pharmacy, University of Arizona, Tucson, AZ 85721, USA; jchien@pharmacy.arizona.edu

**Keywords:** mental health, pain, health care surveys, older adults

## Abstract

Poor mental health is common among older adults with pain, resulting in high economic burden and impaired quality of life. This retrospective, cross-sectional database study aimed to identify characteristics associated with good mental health status among United States (US) adults aged ≥50 years with self-reported pain in the last four weeks using a weighted sample of 2017 Medical Expenditure Panel Survey data. Hierarchical multivariable logistic regression models were used to identify statistically significant predictors of good (versus poor) perceived mental health status. From a weighted population of 57,074,842 individuals, 85.5% (95% confidence interval (CI) = 84.4%, 86.7%) had good perceived mental health. Good mental health was associated most strongly with physical health status (adjusted odds ratio (AOR) = 9.216, 95% CI = 7.044, 12.058). Employed individuals were 1.7 times more likely to report good mental health versus unemployed (AOR = 1.715, 95% CI = 1.199, 2.452). Individuals who had completed less than high school education (AOR = 0.750, 95% CI = 0.569, 0.987) or who reported having a limitation (AOR = 0.513, 95% CI = 0.384, 0.684) were less likely to report good mental health. These key characteristics can be utilized to predict mental health status, which may be investigated to better manage concurrent pain and poor mental health.

## 1. Introduction

The International Association for the Study of Pain (IASP) defines pain as: “an unpleasant sensory and emotional experience associated with, or resembling that associated with, actual or potential tissue damage” [1]. The ambiguous and dynamic nature of pain makes qualifying, quantifying, and managing pain difficult [2], and pain is the highest-reported cause of sought medical attention in the United States (US) [3].

The estimated prevalence of pain in US adults ranges from 100 to 126 million [4,5], and perpetuates in older adults discriminately; the prevalence of pain in older adults (aged >60 years) has been shown to reach rates of 55% [6]. As a result of economic, social, medical, and public health advancements, the percentage of older adults continues to rise in congruency with the number of patients with pain [7]. The total economic cost of pain is estimated to range from USD 560 to USD 635 billion per year in 2010 dollars [4,8]. In addition to economic consequences, pain has been associated with worse health outcomes such as disability, more frequent physician visits, and an overall impaired quality of life [4,9]. Pain is associated with differences in various personal characteristics, including older age [6,10], gender [11,12], ethnicity [5], race [13], socioeconomic class [14], education status [15], employment status [16], comorbidities [17], smoking and alcohol consumption [18,19], and exercise [20,21].

There is a strong, positive association between the physical symptoms of pain and the psychological symptoms, partially explained by related, interlinked neural pathways [22,23]. In addition, patients who report existing physical problems are more likely to report an anxiety/depressive disorder, commit suicide, and smoke cigarettes [23]. Inversely, patients with existing mental health disorders are more likely to report a greater number of physical problems [24]. Pain is also associated with poor mental health regardless of pain etiology [25]. A study by the World Health Organization found patients who reported pain were at a 400% increased disposition to anxiety or depressive disorders, which was consistent across cultures [26]. Despite the discovery of several drug classes that simultaneously manage pain and mental health disorders (serotonin–norepinephrine reuptake inhibitors, anticonvulsants, etc.), pain remains uncontrolled for many, and the prevalence and consequences remain consistently high [4,9,27].

In addition to pharmacological therapy, behavioral and lifestyle modification therapy may be necessary to optimize therapeutic treatments [23,28]. With the likelihood of relapsing and reoccurring mental health disorders, such as depression, increasing in older adults [29], it is important to investigate predictors of mental health status in a variety of populations. In particular, the factors associated with comorbid mental illness and pain in older adults (age ≥ 50) are not well understood. This information is important to minimize the economic and medical consequences arising from non-optimized management of pain and mental health. Therefore, this study sought to identify the predictors of good mental health (versus poor mental health) among a nationally representative sample from the Medical Expenditure Panel Survey (MEPS) dataset of US adults aged ≥50 years with pain in the past four weeks.

## 2. Materials and Methods

### 2.1. MEPS Data and Study Design

MEPS is conducted by the Agency for Healthcare Research and Quality (AHRQ) in multiple interview rounds over a two-year period. MEPS uses a sub-sample of the previous years’ National Health Interview Survey (NHIS) and can produce nationally representative estimates of the non-institutionalized US population by oversampling disabled and minority groups. The MEPS household component (MEPS-HC), one of the key MEPS components, contains self-reported data about each household member surveyed, including (but not limited to): demographic data, health care expenditure and utilization data, health condition data, and health status data [30]. This cross-sectional, retrospective study used the 2017 MEPS full-year consolidated data file (the most current data available at the time of the study) [31]. MEPS respondents provide oral informed consent to voluntarily participate in the survey. The University of Arizona Institutional Review Board approved this study (protocol number 2006721124).

### 2.2. Eligibility

Study participants were included in the analysis if they were alive for the full calendar year, ≥50 years of age, and reported having pain in the last four weeks. Pain was determined based on responses to the question “During the past four weeks, pain interfered with normal work outside the home and housework” of a little bit, moderately, quite a bit, or extremely [32,33].

### 2.3. Dependent Variable

The dependent variable in this study was perceived mental health status, categorized as good or poor. These categories were developed based on responses to the question that asked survey participants to rate their mental health as excellent, very good, good, fair, or poor. For the purposes of this study, responses of excellent, very good, and good were classified as good mental health, and fair or poor as poor mental health [32,33].

### 2.4. Independent Variables

The independent variables in this study were grouped according to Andersen’s Behavioral Model of Health Services Use, as described below [34].

Predisposing factors consisted of: age (in years, 50–64, ≥65); gender (male, female); race (white, other); and ethnicity (Hispanic, non-Hispanic).

Enabling factors consisted of: marital status (married, other); poverty status (<200% federal level, ≥200% federal poverty level); education status (less than high school, completed high school, more than high school); employment status (employed, unemployed); and health insurance status (private, public, uninsured).

Need factors included: limitations (yes, no); number of chronic conditions from the following list: angina, arthritis, asthma, cancer, chronic bronchitis, coronary heart disease, diabetes, joint pain, emphysema, hypercholesterolemia, hypertension, myocardial infarction, other unspecified heart disease, stroke (0, 1, 2, 3, 4, ≥5); pain (little/moderate, quite a bit/extreme); and perceived physical health condition (excellent/very good/good, fair/poor).

External environmental and personal health practice factors were: region (Northeast, Midwest, South, West); regular exercise (yes, no); and smoking status (yes, no) [32,33].

### 2.5. Data Analysis

Using SAS University Edition (SAS institute Inc., Cary, NC, USA), the characteristics of study participants who perceived their mental health to be good with those who perceived their mental health to be poor were compared via chi-square tests. Then, hierarchical logistic regression models were used to assess statistically significant predictors of good perceived mental health status, with poor perceived mental health status serving as the reference group. The first model assessed predisposing factors, and an additional group of characteristics was added to each subsequent model until the final model was reached that included predisposing, enabling, need, and external environmental and personal health practice factors. The a priori alpha level was 0.05.

## 3. Results

This study included a total of 5076 study participants, of which 4225 perceived their mental health as good, while 851 perceived their mental health as poor. This translated to a weighted population of 57,074,842 individuals, of which 85.5% (95% confidence interval (CI) = 84.4%, 86.7%) perceived their mental health as good, while 14.5% (95% CI = 13.5%, 15.6%) perceived their mental health as poor.

The majority of individuals in the study had the following characteristics: aged ≥65 years, female, white race, non-Hispanic, married, ≥200% federal poverty level, completed more than high school education, unemployed, private health insurance coverage, limitations, ≥4 total chronic health conditions, little/moderate pain, excellent/very good/good physical health, no regular exercise, and non-smokers. The most common region was the South. There were significant differences between individuals who reported good mental health and those who reported poor mental health for all characteristics (*p* < 0.05) except age (*p* = 0.8267), gender (*p* = 0.0997), and region (*p* = 0.9556). For further details on the characteristics of study participants, see Table 1.

The strongest predictor of good mental health status was physical health status (adjusted odds ratio (AOR) = 9.216, 95% CI = 7.044, 12.058). Individuals who were employed were approximately 1.7 times more likely to report good mental health than those who were unemployed (AOR = 1.715, 95% CI = 1.199, 2.452). Compared to those who had completed more than high school education, individuals who had completed less than high school education were less likely to report good mental health (AOR = 0.750, 95% CI = 0.569, 0.987). Similarly, compared to individuals who did not report having a limitation, those who did have a limitation were less likely to report good mental health (AOR = 0.513, 95% CI = 0.384, 0.684). The logistic regression model had a c-statistic of 0.844 and a Wald statistic of <0.0001. For further details on the logistic regression results, see Table 2.

## 4. Discussion

While past studies have evaluated the relationship between pain and mental health status, the current study used a nationally representative dataset (i.e., MEPS) to identify predictors of mental health status among older adults with pain. This study identified four statistically significant predictors of mental health status—perceived physical health status, limitation status, employment status, and education level—that demonstrate potential value as investigational, therapeutic lifestyle interventions, for older adults with pain. Such predictive variables may be important not just to help address mental health concerns among older adults with pain, but also emphasize the substantial suicide risk among these patients that must be managed and warrants further research [35,36,37,38,39,40,41,42,43].

### 4.1. Perceived Physical Health Status

Given that pain is a function of physical health status, it seems intuitive that perceived physical health status was associated with mental health status among older adults with pain in this study. Interestingly, in this study, perceived physical health status was the strongest predictor of good mental health status. Previous studies have found similar findings. For example, one study found that poor self-rated health in older adults was associated with “emotional problems”, suggesting correlates of health status may identify patients requiring therapeutic intervention [44]. However, the hypothesized bidirectional association between physical and mental health status may imply that patients with poor physical health require additional preemptive support for the prevention and treatment of mental illness and its secondary complications [22,23]. This is further supported by the inflammation hypothesis, which suggests that mental health decline related to neuroinflammation (facilitated by chronic inflammatory cytokine production), intrinsic to older adults, is exacerbated by pain related to multiple other etiologies [29]. Furthermore, Ohrnberger et al. also found that past physical health was associated with a direct and indirect impact on mental health status—primarily mediated by physical activity [45].

### 4.2. Limitation Status

In the current study, older adults with pain who concurrently reported a limitation were more likely to report poor mental health. Townley et al. found similar findings; patients with a marked limitation status exhibited decreased community participation, demonstrating that barriers to participation may exacerbate reclusiveness and mental illness [46]. Pain (as a surrogate for limitation status) has been shown to serve as a barrier to effective mental health treatment, perpetuating poor mental health [47]. Thielke et al. found that older adults with pain exhibited a diminished response to depression therapy. This raises the question as to whether limitation status is primarily a function of physiology or psychology [48]. While limitation status may be influenced by both, comparing physical therapy (PT) to cognitive behavioral therapy (CBT), Karp et al. found PT to produce a greater response than CBT (47.5% vs. 20.5%) in preventing depression and anxiety in older adults with osteoarthritis [49]. This may suggest the success of the treatment to be closely related to physiologic intervention during cases of comorbid pain and poor mental health. While current and past research reinforce the importance of providing access to rehabilitative care, in older patients with poor mental and physical health status, additional findings of this study also suggest that environmental predictors and modification may promote good mental health status.

### 4.3. Employment Status

The results of this study show that older adults with pain who reported employment were more likely to report good mental health. These findings are consistent with a previous study that found employment was associated with improvements in quality of life and mental health status [47]. This may be in part due to an increased prevalence in daytime sleepiness in unemployed individuals, increasing the risk of pain and poor mental health [50]. Furthermore, past research has found that older adults, especially those who did not have formal education training, when unable to work, were more likely to develop significant out-of-pocket health expenditures and become dependent on others for financial support [51]. In turn, this may lead to feelings of indebtedness and have been associated with a higher susceptibility to developing depression [51]. Future research investigating the financial situation of older adults with pain and poor mental health is warranted. While pain can be limiting to physical activity, creating and providing jobs (that are potentially uninhibited by physical activity) to older adults with pain may support positive mental health by promoting opportunities for socialization, cognitive stimulation, and feelings of value to improve overall quality of life [44,52,53]. However, it is important to recognize an additional potential barrier to employment—education status.

### 4.4. Education Status

The current study also found that older adults with pain, who also did not complete a high school level education, were associated with a greater likelihood of reporting poor mental health. Previous research has found that educational attainment and early education have significant positive neuropsychological benefits, which prevent cognitive decline [54]. Furthermore, this may indicate that older adults may require additional educational opportunities for improving overall knowledge and health literacy. Poor health literacy may arise from a limited education status and promote a poor understanding of self-care and pain management. Another study found that poor health literacy was associated with both poor physical and mental health [55]. Ultimately, limited education may perpetuate poor health literacy, mistreated pain, and poor mental health.

### 4.5. Limitations and Future Work

The limitations of this study include those intrinsic to self-reported and secondary data analysis, in particular recall bias from survey participants, although the frequent MEPS interviews (reoccurring every 4 to 5 months) help reduce this risk. The study design was unable to determine a cause-and-effect relationship yet was able to demonstrate a statistical association between four predictor variables and mental health status. The strength of the study was the adjusted MEPS design, which provided a large nationally representative sample for analysis and enhanced generalizability of the findings. Based on the findings of this study, future research should investigate whether interventions to address the four factors associated with mental health status can lead to changes in mental health status. This may involve a longitudinal analysis to determine the trajectory of comorbid pain and mental health status.

## 5. Conclusions

In summary, this is the first study to evaluate predictors of mental health status in older adults on a national level using data from the 2017 MEPS dataset. Four statistically significant predictors of mental health status were found: [1] perceived physical health status, [2] limitation status, [3] employment status, and [4] education level. Positive physical health status was found to have the strongest level of association with positive mental health. These findings indicate important characteristics to address to help improve mental health status among older adults with pain, which may help emphasize a shift to predictive medicine rather than secondary prevention for mental health conditions. There is a need for future longitudinal studies in order to determine causality.

## Figures and Tables

**Table 1:** Characteristics of older United States adults (age ≥ 50 years) with self-reported pain in the past four weeks, stratified by good and poor perceived mental health status.

Factors	Good Perceived Mental Health Status (Weighted N = 48,820,087) Weighted Percent (95% Confidence Interval)	Poor Perceived Mental Health Status (Weighted N = 8,254,755) Weighted Percent (95% Confidence Interval)	*p*
Predisposing:			
Age (years)			0.8267
50–64	85.4 (83.9–86.9)	14.6 (13.1–16.1)	
≥65	85.7 (84.0–87.4)	14.3 (12.6–16.0)	
Gender			0.0997
Male	86.5 (84.9–88.1)	13.5 (11.9–15.1)	
Female	84.8 (83.3–86.2)	15.2 (13.8–16.7)	
Race			0.0004
White	86.5 (85.2–87.7)	13.5 (12.3–14.8)	
Other	81.6 (79.1–84.1)	18.4 (15.9–20.9)	
Ethnicity			0.0004
Hispanic	80.4 (77.0–83.7)	19.6 (16.3–23.0)	
Non-Hispanic	86.1 (84.9–87.3)	13.9 (12.7–15.1)	
Enabling:		
Marital status			<0.0001
Married	88.1 (86.7–89.6)	11.9 (10.4–13.3)	
Other	82.1 (80.3–83.9)	17.9 (16.1–19.7)	
Poverty status			<0.0001
<200% federal level	75.6 (73.3–77.9)	24.4 (22.1–26.7)	
≥200% federal level	90.3 (88.9–91.6)	9.7 (8.4–11.1)	
Education status			<0.0001
Less than high school	75.1 (71.8–78.5)	24.9 (21.5–28.2)	
Completed high school	85.2 (83.4–86.9)	14.8 (13.1–16.6)	
More than high school	89.4 (87.9–90.8)	10.6 (9.2–12.1)	
Employment status			<0.0001
Employed	93.7 (92.4–95.1)	6.3 (4.9–7.6)	
Unemployed	80.3 (78.6–82.0)	19.7 (18.0–21.4)	
Health insurance status			<0.0001
Private	91.3 (90.1–92.6)	8.7 (7.4–9.9)	
Public	75.9 (73.5–78.2)	24.1 (21.8–26.5)	
Uninsured	82.1 (75.4–88.7)	17.9 (11.3–24.6)	
Need:		
Limitation			<0.0001
Yes	77.5 (75.5–79.5)	22.5 (20.5–24.5)	
No	93.8 (92.8–94.8)	6.2 (5.2–7.2)	
Number of chronic conditions			<0.0001
0	93.0 (89.8–96.2)	7.0 (3.8–10.2)	
1	90.7 (88.0–93.3)	9.3 (6.7–12.0)	
2	90.0 (87.5–92.5)	10.0 (7.5–12.5)	
3	88.4 (86.0–90.7)	11.6 (9.3–14.0)	
4	85.8 (83.1–88.6)	14.2 (11.4–16.9)	
≥5	79.1 (76.9–81.3)	20.9 (18.7–23.1)	
Pain			<0.0001
Little/moderate	90.2 (89.1–91.3)	9.8 (8.7–10.9)	
Quite a bit/extreme	71.5 (68.6–74.4)	28.5 (25.6–31.4)	
Perceived physical health status			<0.0001
Excellent/very good/good	95.4 (94.5–96.2)	4.6 (3.8–5.5)	
Fair/poor	58.9 (55.7–62.1)	41.1 (37.9–44.3)	
External environmental and personal health practices:		
Region			0.9556
Northeast	85.4 (83.3–87.6)	14.6 (12.4–16.7)	
Midwest	85.7 (83.0–88.4)	14.3 (11.6–17.0)	
South	85.2 (83.4–87.0)	14.8 (13.0–16.6)	
West	86.1 (83.3–88.8)	13.9 (11.2–16.7)	
Regular exercise			<0.0001
Yes	89.9 (88.5–91.3)	10.1 (8.7–11.5)	
No	82.4 (80.7–84.1)	17.6 (15.9–19.3)	
Smoking status			<0.0001
Yes	78.9 (75.7–82.2)	21.1 (17.8–24.3)	
No	86.8 (85.5–88.0)	13.2 (12.0–14.5)	

Analysis based on 5076 (un-weighted) United States adults alive during the calendar year 2017, age ≥ 50 years, with self-reported pain in the past four weeks. Good mental health (un-weighted n = 4225) was defined as a response of excellent, very good, or good, while poor mental health (un-weighted n = 851) was defined as a response of fair or poor when asked about perceived mental health status. Differences between groups were assessed using chi-square tests.

**Table 2:** Predictors of good perceived mental health status among older United States adults (age ≥ 50 years) with self-reported pain in the past four weeks.

Factors	Adjusted Odds Ratio	95% Confidence Interval
Predisposing:		
Age (years)		
50–64 vs. ≥65	0.818	0.621–1.077
Gender		
Male vs. female	1.141	0.919–1.417
Race		
White vs. other	1.132	0.890–1.440
Ethnicity		
Hispanic vs. non-Hispanic	0.807	0.614–1.062
Enabling:		
Marital status		
Married vs. other	0.877	0.700–1.098
Poverty status		
<200% vs. ≥200% federal level	0.794	0.619–1.017
Education status		
Less than high school vs. more than high school	**0.75**	**0.569**–**0.987**
Completed high school vs. more than high school	1.028	0.808–1.307
Employment status		
Employed vs. unemployed	**1.715**	**1.199**–**2.452**
Health insurance status		
Private vs. uninsured	1.376	0.686–2.762
Public vs. uninsured	0.896	0.437–1.837
Need:		
Limitation		
Yes vs. no	**0.513**	**0.384**–**0.684**
Number of chronic conditions		
0 vs. ≥5	0.938	0.483–1.822
1 vs. ≥5	0.683	0.431–1.083
2 vs. ≥5	1.102	0.742–1.636
3 vs. ≥5	0.922	0.670–1.268
4 vs. ≥5	0.892	0.646–1.230
Pain		
Little/moderate vs. quite a bit/extreme	1.121	0.875–1.435
Perceived physical health status		
Excellent/very good/good vs. fair/poor	**9.216**	**7.044**–**12.058**
External environmental and personal health practices:		
Region		
Northeast vs. West	0.955	0.664–1.374
Midwest vs. West	0.923	0.624–1.366
South vs. West	1.19	0.851–1.665
Regular exercise		
Yes vs. no	1.146	0.917–1.433
Smoking status		
Yes vs. no	0.875	0.662–1.157

Analysis based on 5076 (un-weighted) United States adults alive during the calendar year 2017, age ≥ 50 years, with self-reported pain in the past four weeks. Good mental health (un-weighted n = 4225) was defined as a response of excellent, very good, or good, while poor mental health (un-weighted n = 851; reference group) was defined as a response of fair or poor when asked about perceived mental health status. Bold indicates the variable was a predictor of perceived health status.

## Data Availability

The data presented in this study are available on request from the corresponding author.
